# Task decomposition: a framework for comparing diverse training models in human brain plasticity studies

**DOI:** 10.3389/fnhum.2013.00640

**Published:** 2013-10-08

**Authors:** Emily B. J. Coffey, Sibylle C. Herholz

**Affiliations:** ^1^Montreal Neurological Institute, McGill UniversityMontreal, QC, Canada; ^2^International Laboratory for Brain, Music and Sound Research, Université de MontrealMontreal, QC, Canada; ^3^German Center for Neurodegenerative Diseases (DZNE)Bonn, Germany

**Keywords:** expertise, plasticity, training, MRI, multisensory learning

## Abstract

Training studies, in which the structural or functional neurophysiology is compared before and after expertise is acquired, are increasingly being used as models for understanding the human brain’s potential for reorganization. It is proving difficult to use these results to answer basic and important questions like how task training leads to both specific and general changes in behavior and how these changes correspond with modifications in the brain. The main culprit is the diversity of paradigms used as complex task models. An assortment of activities ranging from juggling to deciphering Morse code has been reported. Even when working in the same general domain, few researchers use similar training models. New ways to meaningfully compare complex tasks are needed. We propose a method for characterizing and deconstructing the task requirements of complex training paradigms, which is suitable for application to both structural and functional neuroimaging studies. We believe this approach will aid brain plasticity research by making it easier to compare training paradigms, identify “missing puzzle pieces,” and encourage researchers to design training protocols to bridge these gaps.

## INTRODUCTION

The idea that the structure and function of the human brain remains somewhat open to alteration by experience over the lifespan is now well established (Wan and Schlaug, 2010; Zatorre et al., 2012), although researchers have not yet formed a comprehensive view of how – and under which conditions – this occurs.

In this paper, we focus on the research looking at training-related plasticity in human subjects that uses complex skills as models, such as juggling (e.g., Draganski et al., 2004; Boyke et al., 2008; Scholz et al., 2009), golfing (e.g., Bezzola et al., 2011), or various aspects of making music (e.g., Lappe et al., 2008; Hyde et al., 2009). Work using such skills complements earlier and ongoing research on more basic aspects of brain–behavior relationships, such as learning a simple finger-tapping task (e.g., Ungerleider et al., 2002).

Complex tasks offer several advantages over simpler tasks, as models: they involve more ecologically valid learning experiences; they offer an opportunity to study higher-order and domain-general aspects of learning; they are often inherently interesting to subjects, which offers benefits particularly for longitudinal studies in motivation and compliance; and most significantly, recent evidence suggests that the multisensory and sensorimotor nature of such tasks is particularly effective in inducing plasticity both in sensory and association cortical areas (Lappe et al., 2008; Paraskevopoulos et al., 2012).

The complex nature of the tasks also introduces major challenges for the comparison and integration of results across training studies. These studies usually produce complex results, including changes in activity or structure in many different brain regions. Strictly speaking, only a direct comparison can demonstrate specificity of plastic changes. Rarely are these available, though; the majority of training studies have used either control groups without training, or comparison with a within-subject baseline to assess the possibility of developmental or other non-specific changes. In a recent review of 20 studies on the structural effects of a range of cognitive and multisensory training paradigms, for example, only three compared the task of interest with a second task (Thomas and Baker, 2013). Since few direct comparisons are available, inferences regarding the specificity of task effects rest on arguments about the relevance of the brain structure to apparently related tasks, or on correlational evidence in the form of relationships with behavioral change. Typically, this works well when outcomes can be predicted beforehand, and indeed training studies report changes in brain areas (or other physiological measurements) that are known from previous work to be involved in related activities (e.g., in auditory and motor areas for musical training; Hyde et al., 2009). Findings which are not predicted, for example because relationships of higher-order cognitive systems to training are yet unknown, can pose greater interpretation problems. This is due to the dissimilarity of the studies available for comparison – studies using unalike paradigms offer only very weak and caveated support for one another.

As well as for explaining unexpected results, the diversity of complex task paradigms makes it hard for researchers to draw general bigger-picture conclusions about brain plasticity from the aggregate results. As Erickson (2012) notes, “it is difficult to retrieve much homogeneity in the outcomes from such a heterogeneous set of studies and primary aims.” There are basic questions about brain plasticity which remain unresolved. For example, why does acquiring some skills lead to increases in measures of brain structure or activity (generally interpreted as strengthening existing capacity or recruiting additional machinery), whereas others lead to decreases (generally interpreted as improved efficiency and requiring less processing effort)? What determines whether a skill is transferable to other behaviors or results in a highly specific behavioral gain? It will be difficult to piece together answers from such a heterogeneous group of studies.

In sum, complex tasks are problematic because their study design space is vast. Because neuroimaging studies such as these are resource-intensive, particularly longitudinal studies which allow us to test causal hypotheses directly, systematically varying each aspect of the training paradigms is an impractical solution. The wide and sparse coverage of potential complex tasks that is already represented in literature implicates nearly every cognitive system, and in various combinations. Even when studies ostensibly use similar tasks, they may differ on tens of potentially important training design parameters, among them the control condition used (i.e., none, between subjects, or within subjects), the sample size, population characteristics, the duration and intensity of training, and the subjects’attained proficiency. Concurrently, rapid evolution of neuroimaging and analysis methods further reduce the comparability of studies.

One might argue for a return to simpler training paradigms until basic mechanisms of plasticity are more fully understood, were it not for the fact that a better understanding of complex task training-related plasticity and its underlying mechanisms is needed now. Important motivations fuelling the observed increase in research comes from promising yet early attempts to improve neurological rehabilitation after injury or stroke (Altenmüller et al., 2009), to prevent of cognitive decline in old age (Wan and Schlaug, 2010), to develop auditory training tools that target the brain to treat auditory processing disorder (Musiek et al., 2002; Loo et al., 2010), and possibly to transform the way the effectiveness of therapeutics and training techniques is evaluated (Erickson, 2012).

We must find new ways in which to integrate knowledge generated using many models. In the remainder of this paper, we propose one such approach.

## A FRAMEWORK TO CHARACTERIZE TRAINING TASKS

In professional environments in which training of personnel must be both effective and efficient, instructional designers have refined the art of training; i.e., producing trainees with specific skills and knowledge. Briefly, one such instructional design process known as the Dick and Carey Systems Approach Model (Dick et al., 2004) begins with the definition of a set of concrete goals called “performance objectives” (POs). Flow charts are then used to illustrate the analysis of complex activities into smaller activities or functions. The POs and task breakdown serve as a reference when designers create evaluation measures, define an appropriate instructional strategy, develop and select instructional materials, and finally, evaluate the effectiveness of the training.

Whereas the goal of instructional designers is the successful and measurable transmission of skills and knowledge, the goals of researchers are usually either to design a training paradigm which provokes change in a certain brain structure or function, or to better understand what changes might have been caused by an existing training paradigm or naturalistic learning experience. In either case, two ideas can be borrowed from instructional design; the use of POs, and the task analysis. These are useful both when designing studies and when evaluating existing designs for comparability.

## PERFORMANCE OBJECTIVES

**FIGURE 1:**
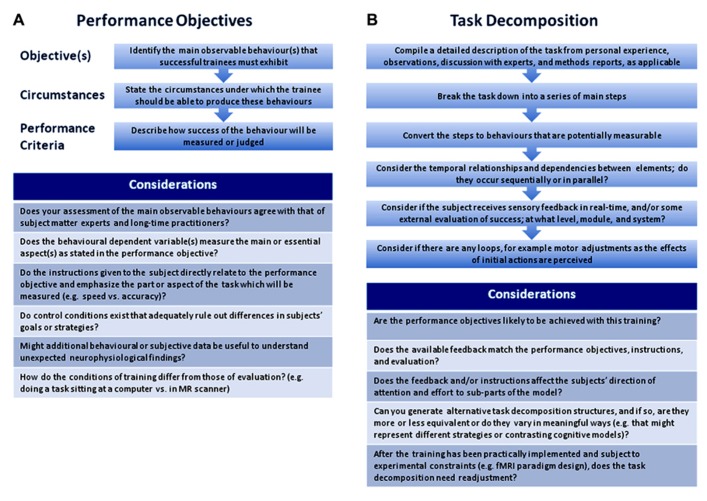
**Suggested steps and considerations for writing performance objectives (A) and for task decomposition (B)**.

A PO consists of a description of the desired outcome behavior; the circumstances under which the outcome should be met including any equipment, instructions, environmental variables like condition of the subjects and availability of feedback or coaching; and the criteria used to judge the learner’s performance. It is worthwhile to create POs for a training study because they help researchers to maintain coherence between the performed task, the subjects’instructions, and the behavioral-dependent variables, and to consider addressing possible alternative explanations with additional controls or measures. In **Figure 1A**, we include some suggestions for writing and using POs.

The POs for some training studies are relatively straightforward and can be easily deduced from the methods description. For example, a recent study (Landi et al., 2011) investigated structural changes associated with motor adaptation. The PO for this task could have been written as follows:

**• Objective:** Minimize the average target–cursor distance over the session.

**• Circumstances:** Controlling a joystick using the thumb and index fingers of the right hand to follow a moving target, while a complex perturbation is applied.

**• Criteria of performance:** The distance between target and cursor averaged for each block and expressed as a percentage of the baseline.

Extracting POs from other training studies, particularly ones involving naturalistic designs on leisure activities, is sometimes less straightforward because the tasks are not always comprehensively described. This might be because a detailed account of a very popular activity seems unnecessary, because the training is not strictly under the experimenter’s control, or because the aim of a study might be only to show that any change was caused by doing some activity.

In the study reports, conclusions are nevertheless almost always drawn about the relationships between many specific physiological findings and possible task-relevant cognitive activity. The details of how real-life complex tasks are taught and learned may be relevant for this interpretation. For example, a novice violinist who is encouraged to play entirely by ear will exercise a different set of cognitive skills than one who is learning by reading musical notation, which could explain differences in activity in visual and auditory areas. Making a clear statement about the intended focus of the training early in the study design phase makes it easier to identify supplementary measures and controls that might have explanatory value (e.g., a post-practice questionnaire to provide insight into the instructor’s strategy).

## TASK DECOMPOSITION

Task analysis has a long tradition in cognitive psychology and behaviorism where it has been applied to develop models for behavioral contingencies (e.g., Skinner, 1954), to create computational models (Newell and Simon, 1972), to analyze individual differences in reasoning (Sternberg, 1977), and to build computer models of cognitive architecture (Anderson et al., 2003, 2009; Qin et al., 2003).

Unlike previous work in which creating models of behavior and cognition was the goal, our motivation is to be able to compare the neuroplasticity results from multifaceted tasks and from researchers of different theoretical persuasions. We must therefore target a level of generality that can be linked to the functional networks and modules accessible to neuroimaging methods, rather than on finer-grained analyses, such as specific thought processes. We must also prioritize training-related changes, and we must try to remain as theoretically neutral as possible, such that two researchers studying complex tasks need not first agree on cognitive and mechanistic models for each of many task components.

We propose a “task decomposition” in which a complex task is broken down into elements that are necessary to achieve the PO (see **Figure 1B** for suggested steps). To facilitate agreement and limit inherent theoretical assumptions, we suggest that the elements be formulated as potentially measurable behaviors (e.g., hold a sequence of notes in mind) rather than as cognitive constructs (e.g., auditory working memory). Choices must be made as to the generality of the elements, for example, whether a hand movement is broken down into finger movements. This will depend on the ability of the experimental design to resolve smaller elements, but if modeled hierarchically, elements could be expanded or collapsed to different levels of detail to accommodate different comparative goals. A common taxonomy of behavioral elements would aid task comparison. To the best of our knowledge a suitable taxonomy does not exist, but one readily observes multiple reoccurring elements when working through several decompositions. Enumerating and standardizing the wording of these would be a necessary step for any meta-analysis.

We relate the elements temporally, which is straightforward and does not introduce many cognitive assumptions. Elements may occur in sequence or concurrently, and series of events may occur as a discrete unit or as a loop. We have included behaviors normally considered both lower and higher-order cognition as elements (e.g., visual observation vs. evaluating the success of an action). Metacognitive elements like the selection of different strategies could also be included, but this would add a level of complexity, for example, if one evaluation element switched between two possible structures. It should first be considered if the component is a focus of neuroplasticity; i.e., likely to have changed with the training in a way that was measurable.

For some tasks, the selection of elements and their arrangement may lead to several competing structures which represent neurophysiologically relevant differences. In the case of a longitudinal study in which transient effects are observed over several measurement points (e.g., Taubert et al., 2010), different structures could usefully be related to expertise acquisition. Different structures could be caused by incorrect assumptions or inter-individual differences; we believe that even in these cases it will be valuable to document the task as a basis for discussion – problems can then be resolved empirically.

In the following section, we illustrate how task decomposition might be used to compare two tasks. We focus on multisensory training tasks in this paper, but this approach might also be applied to more purely cognitive training (see for example reviews by Buschkuehl et al., 2012; Guida et al., 2012).

## AN EXAMPLE OF USING TASK DECOMPOSITION

Using this approach, we start to explore how changes in gray matter concentration as measured by voxel-based morphometry are related to two training tasks; visuomotor tracking described previously (Landi et al., 2011) and 40 h of amateur-level golf practice as an uncontrolled leisure activity (Bezzola et al., 2011). The conclusions we can draw from this two-task analysis might seem trivial as it is not difficult to compare two studies without decomposition. However, our goal here is to offer examples of task decomposition diagrams (TDDs) and a simple illustration of the principle of using them to compare tasks.

**FIGURE 2:**
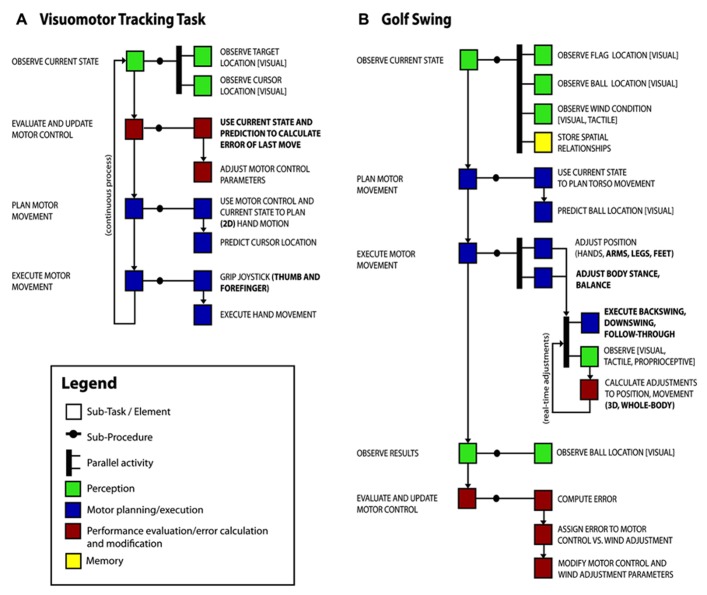
**Task decomposition diagrams for two training paradigms, (A) the visuomotor tracking task of Landi et al. (2011), and (B) a golf swing which we presume was a major part of golf training (Bezzola et al., 2011).** Sub-tasks of the main activity are shown with boxes. We have grouped similar elements into classes for the purposes of visualization (perception – green, motor – blue, evaluation/error calculation – red, memory – yellow), though the elements themselves are likely to be more useful for task comparison. Arrows show dependencies between sub-tasks and thick bars indicate concurrent activities. Components that differ between the visuomotor tracking task and the golf swing are in bold font.

We have prepared a possible task decomposition for each study (see **Figure 2**) and highlighted the elements that differ between the tasks (bold font). We expect to find similar neurophysiological changes in tasks that share a component, and no change in this area with other tasks that do not have this component or do not stress adaptation and learning of this component.

The PO of the visuomotor tracking task was to minimize the average target–cursor distance over the session, whereas the PO of the golf practice was presumably to execute a golf swing so as to move ball to target location. The TDDs show overlap of task requirements relating to motor planning and execution. These can account for the convergent findings of changes in motor areas in the dorsal stream encompassing primary motor cortex (M1) contra-lateral to the (most) trained hand in both studies. In contrast, the divergence of the tasks in some aspects, in particular visuomotor control in two vs. three dimensions, hand vs. full body action including balance, and a tight coupling of action and outcome vs. integration of several separate movements into a larger sequence that involves more planning, could account for a discrepancy in findings in the frontal and parietal association areas, as these areas have been shown to be related to planning action sequences and visuomotor integration (Andersen and Buneo, 2002; Molnar-Szakacs et al., 2006) and the representation of one’s own body in spatial reference frames (Vallar et al., 1999) – elements that are important parts of golf, but not visuomotor tracking.

This sort of analysis could then be used to investigate explanatory hypotheses. For example, based on a previous functional magnetic resonance imaging (fMRI) study using the same task (Della-Maggiore and McIntosh, 2005), Landi et al. (2011) had expected changes in a network including M1, posterior parietal cortex (PPC), and cerebellum, but found only the M1 result. Cerebellum and PPC are relevant functionally for online error correction, but the lack of structural changes might be due to the similarity of the manual tracking task to everyday tasks in these respects. The more novel kinds of whole-body and multisensory error corrections that are necessary for learning golf swings might stimulate greater neurophysiological adaptation. Next steps might be to compare these results with those from other manual tasks with these error correction requirements, or to design one.

## OTHER APPLICATIONS AND CAUTIONS

Beyond uncovering patterns of task demands and neurophysiological effect across training studies, task decomposition could be used in other ways in plasticity research. Characterizing tasks used in human and animal research could facilitate cross-field comparisons from systems to circuit level (e.g., Sagi et al., 2012). Hypotheses as to the cause for divergent empirical findings can be tested by designing tasks in which only those sub-components suspected of causing the change are manipulated. It would also be possible to start with a brain region of interest, identify common characteristics or components of trainings that lead to enhancements, and design rehabilitative tasks emphasizing those elements.

There are several possible pitfalls to this approach: *post hoc* models could be biased toward task components that have known neural correlates that are in line with the results of the study; omitting crucial task components due to oversight or bias might result in incorrect assignment of neuroimaging results to task components that are included in the model; and since most brain regions are involved in multiple, different cognitive processes, changes in the same brain region may be due to different task components depending on context. These challenges parallel challenges interpreting neuroimaging data in general (e.g., Vul et al., 2009; Simmons et al., 2011), and can be partially addressed by *a priori* model setup and awareness of these limitations during interpretation.

## CONCLUSION

In the rapidly evolving field of training-related plasticity, integration of results across studies will be crucial. For this, an approach like task decomposition could be useful to disentangle the respective influences of task demands on neuroplasticity, and increase the informational value and impact of each resource-intensive training study. By integrating across studies, we will be able to reveal specific and general mechanisms of plasticity within and across modalities such as the motor, visual, and auditory systems, and enhance our understanding of the role of higher-order functions and association areas in cortical plasticity. We argue that if researchers systematically consider what sub-tasks participants must perform in order to achieve training goals, and communicate them in the literature along with other aspects of their study design, it may turn the diversity in training studies into an advantage rather than an impediment by allowing us to extract meaning from aggregate results and to target future studies efficiently.

## Conflict of Interest Statement

The authors declare that the research was conducted in the absence of any commercial or financial relationships that could be construed as a potential conflict of interest.
